# Myocardial mechanics in dilated cardiomyopathy: prognostic value of left ventricular torsion and strain

**DOI:** 10.1186/s12968-021-00829-x

**Published:** 2021-12-02

**Authors:** Andreas Ochs, Johannes Riffel, Marco M. Ochs, Nisha Arenja, Thomas Fritz, Christian Galuschky, Andreas Schuster, Oliver Bruder, Heiko Mahrholdt, Evangelos Giannitsis, Norbert Frey, Hugo A. Katus, Sebastian J. Buss, Florian André

**Affiliations:** 1 Department of Cardiology, Angiology and Pneumology, University of Heidelberg, Im Neuenheimer Feld 410, 69120 Heidelberg, Germany; 2 DZHK (German Centre for Cardiovascular Research), Partner Site Heidelberg, Heidelberg, Germany; 3 Department of Cardiology, Solothurner Spitäler AG, Kantonsspital Olten, Olten, Switzerland; 4 TomTec Imaging Systems GmbH, Unterschleissheim, Germany; 5 Herzzentrum, Göttingen, Germany; 6 Contilia Herz- und Gefäßzentrum, Essen, Germany; 7 Robert-Bosch-Krankenhaus, Stuttgart, Germany

**Keywords:** Dilated cardiomyopathy, Cardiac magnetic resonance, Strain, LV torsion, Prognosis

## Abstract

**Background:**

Data on the prognostic value of left ventricular (LV) morphological and functional parameters including LV rotation in patients with dilated cardiomyopathy (DCM) using cardiovascular magnetic resonance (CMR) are currently scarce. In this study, we assessed the prognostic value of global longitudinal strain (GLS), global circumferential strain (GCS), global radial strain (GRS) and LV torsion using CMR feature tracking (FT).

**Methods:**

CMR was performed in 350 DCM patients and 70 healthy subjects across 5 different European CMR Centers. Myocardial strain parameters were retrospectively assessed from conventional balanced steady-state free precession cine images applying FT. A combined primary endpoint (cardiac death, heart transplantation, aborted sudden cardiac death) was defined for the assessment of clinical outcome.

**Results:**

GLS, GCS, GRS and LV torsion were significantly lower in DCM patients than in healthy subjects (all p < 0.001). The primary endpoint occurred in 59 (18.7%) patients [median follow-up 4.2 (2.0–5.6) years]. In the univariate analyses all strain parameters showed a significant prognostic value (p < 0.05). In the multivariate model, LV strain parameters, particularly GLS provided an incremental prognostic value compared to established CMR parameters like LV ejection fraction and late gadolinium enhancement. A scoring model including six categorical variables of standard CMR and strain parameters differentiated further risk subgroups.

**Conclusion:**

LV strain assessed with CMR FT has a high prognostic value in patients with DCM, surpassing routine and dedicated functional parameters. Thus, CMR strain imaging may contribute to the improvement of risk stratification in DCM.

**Supplementary Information:**

The online version contains supplementary material available at 10.1186/s12968-021-00829-x.

## Background

As the assessment of myocardial deformation provides incremental prognostic value in heart failure [1, 2], a better understanding of myocardial mechanics in dilated cardiomyopathy (DCM)—including left ventricular (LV) rotational mechanisms—may improve risk stratification and facilitate treatment guidance.

Cardiovascular magnetic resonance (CMR) allows for the assessment of cardiac morphology and function with high spatial resolution and an excellent intrinsic blood-to-tissue contrast [3]. It is regarded as the current reference standard for the evaluation of LV morphology and function [4, 5]. For strain analysis in CMR, feature tracking (FT) has been established as a useful technique as it allows for the retrospective analysis of conventional balanced steady-state free precession (bSSFP) series without the need for additional, dedicated CMR strain sequences [6, 7]. CMR FT strain measurements feature reliable results showing an adequate agreement with echocardiography and CMR tagging [8–11]. Additionally, reference values have been published for adults and children [12–14].

Previous studies showed, that global longitudinal strain (GLS), global circumferential strain (GCS), global radial strain (GRS) and LV torsion are altered in various cardiomyopathies [15, 16]. Although the prognostic value of myocardial strains was shown in prior studies, data on the prognostic value of the different strain parameters—and especially LV torsion—in DCM patients are heterogenous and scarce [15, 17, 18].

Therefore, the aim of this study was to assess GLS, GCS, GRS and LV torsion in a large, multicenter cohort of DCM patients, and to evaluate their prognostic value.

## Methods

### Study subjects

Diagnosis of DCM was based on the 1995 World Health Organization/International Society and Federation of Cardiology criteria [19]. Inclusion criteria were an impaired systolic function with LV ejection fraction (LVEF) ≤ 45% and dilated LV end-diastolic diameter (LVEDD) obtained from another imaging modality than CMR, the absence of relevant coronary artery disease (defined as ≥ 50% luminal stenosis), myocardial infarction or coronary revascularization, significant valvular disease, hypertensive heart disease and congenital heart defects.

A population of 410 patients with non-ischemic DCM from University Hospital Heidelberg and the EuroCMR Registry was screened. In 45 patients the minimum interval of one year for follow-up data was not reached resulting in exclusion. Another 15 subjects were excluded due to insufficient tracking quality. Finally, a study population of 350 DCM patients was retrospectively included in the study. 306 patients derive from University Hospital Heidelberg, the 44 patients of the EuroCMR Registry from four different imaging centers in Switzerland, Belgium and Lithuania.

In addition, 70 subjects drawn from a study population of proven healthy subjects were analysed at University Hospital Heidelberg. They all underwent a strict selection process including detailed medical history, physical examination, comprehensive blood tests, 12-lead electrocardiogram (ECG) and stress CMR. An oral glucose tolerance test was performed to exclude impaired glucose tolerance. Subjects with a history, signs or symptoms of a cardiac disease were excluded. Additional exclusion criteria were arterial hypertension, cerebrovascular or other relevant diseases, a regular intake of drugs except for thyroid or contraceptive drugs or vitamins.

All participants gave written informed consent and the study was approved by the local ethics committee. A part of the study population was already part of prior CMR trials [15, 20].

### Image acquisition and analysis

CMR examinations at the University Hospital Heidelberg were performed on a 1.5T clinical scanner (Achieva^®^, Philips Healthcare, Best, the Netherlands). Subjects identified from the EuroCMR Registry were examined at various 1.5T and 3T CMR scanners from Philips Healthcare (Intera^®^, Ingenia^®^, Achieva^®^) and Siemens Healthineers (Erlangen, Germany; Aera^®^, Avanto^®^, Skyra^®^, Verio^®^). Image acquisition was performed in accordance with the Society for Cardiovascular Magnetic Resonance (SCMR) guidelines to allow for data pooling [21].

Analyses of all subjects for ventricular volumes, LVEF and other morphological and functional parameters were derived from short axis and long axis views on commercially available workstations (Viewforum^®^ and IntelliSpace Portal^®^, Philips Healthcare) and dedicated CMR software (cvi42™, Circle Cardiovascular Imaging, Calgary, Alberta, Canada) at our imaging center from different examiners with many years of expertise in CMR. All ‘standard’ morphological and functional parameters including late gadolinium enhancement (LGE) were analyzed in accordance with actual recommendations [22]. Papillary muscles were part of the LV volume. LGE was acquired in all DCM patients. LGE sequences were routinely interpreted by experienced physicians with level 3 CMR certification of the German Cardiac Society or comparable qualification. LGE extent was not quantified due to the mostly diffuse demarcation of myocardial fibrosis. Every LGE pattern was considered—except for mild, unspecific LGE at the basal right ventricle (RV) insertion.

In EuroCMR Registry patients, baseline parameters as well as anthropometric data were provided by the respective participating centre and retrieved from the EuroCMR Registry database.

### Data analysis

**Fig. 1:**
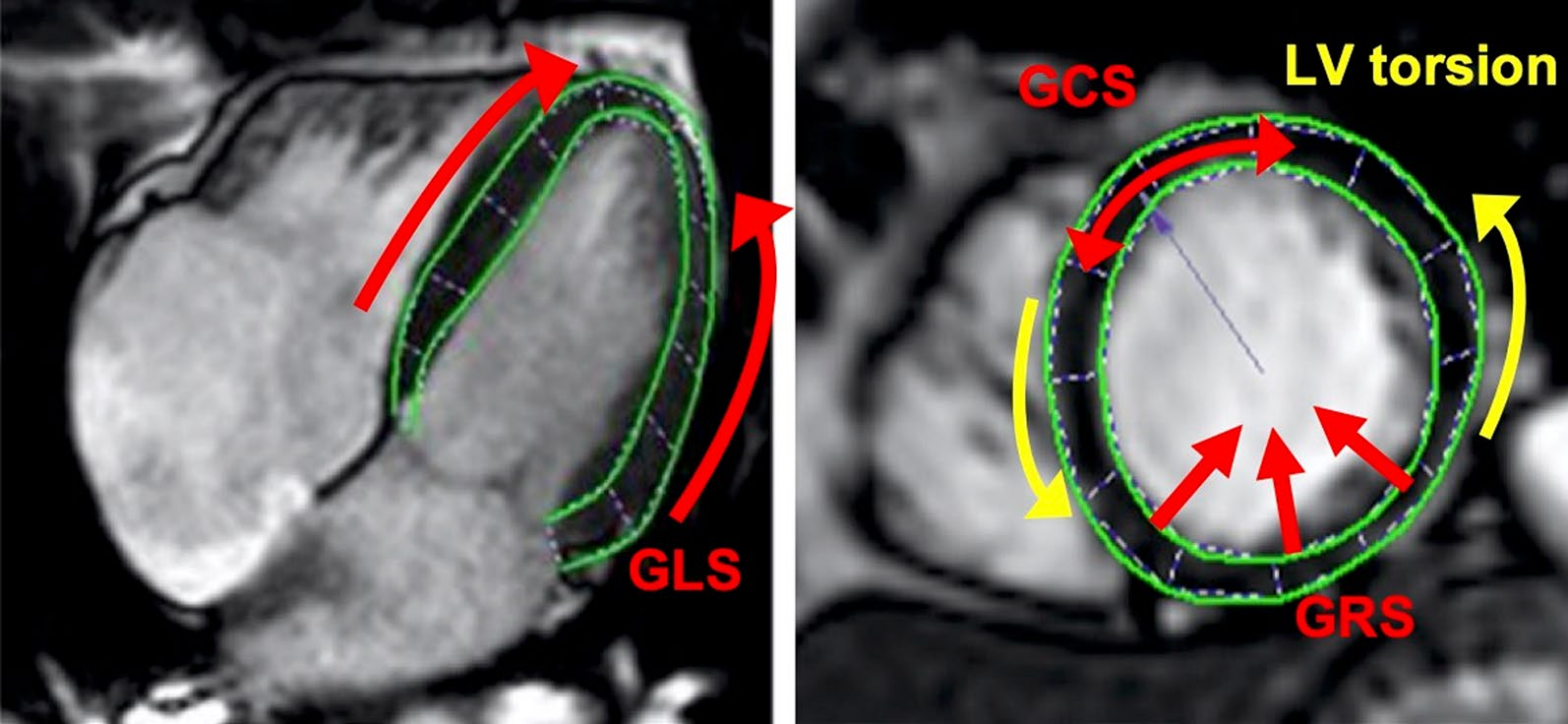
Illustration of the assessed strain parameters in long and short axis views. Long axis views (left: 4-chamber view) were used for the measurement of global longitudinal strain (GLS), three short axis views at apical, midventricular and basal level (right: apical short axis view) were used to evaluate global circumferential strain (GCS), global radial strain (GRS) and left ventricular (LV) torsion

Strain analysis was performed on bSSFP cine images using dedicated 2D CMR FT software (TomTec Imaging Systems GmbH, Unterschleissheim, Germany) at the core lab of the University of Heidelberg. LV endocardial and epicardial borders were manually drawn at end-diastole and the tracking of the myocardium over the whole cardiac cycle was automatically conducted. All measurements were repeated three times [23]. If necessary, the contouring was manually optimized. Patients were excluded if no satisfying myocardial tracking was achievable. Long axis views (2-chamber, 3-chamber, and 4-chamber views) were used for the measurement of GLS, three short axis views at apical, midventricular and basal level were used to evaluate GCS, GRS and LV rotation (Fig. 1). In our study GLS, GCS, GRS and LV rotation of apex and base were calculated using the following approach [15]: First, the three measurements were averaged for every segment resulting in segmental mean curves. Second, the peak of a global mean curve (average of all segmental mean curves over the whole cardiac cycle) was used as the global strain value.

Apical and basal slices at standardized 25% and 75% levels were selected for the quantification of rotational parameters, as this approach provides best reproducibility [10]. As viewed from the apex, ‘normal’ LV rotation was defined as a counterclockwise systolic rotation of the apex and a clockwise rotation of the base. LV rotation was expressed as LV twist and LV torsion. The peak difference of LV apical and basal rotation at the same point of the cardiac cycle was defined as LV twist, whereas the LV torsion was calculated as the relation of LV twist/LV length. LV length was defined as the distance between the apical and basal short axis slices used for LV rotation analyses. All rotational parameters were measured at ‘mid-wall’.

### Follow-up data and definition of study endpoints

Physicians, blinded to the CMR results, contacted each patient or an immediate family member to obtain follow-up data. Cardiac death, heart transplantation and aborted sudden cardiac death (SCD) by appropriate implanted cardioverter defibrillator (ICD) discharge (including successful antitachycardia pacing = ATP) due to ventricular tachycardia or ventricular fibrillation were defined as the combined *primary endpoint*. The events of the primary endpoint and hospitalization due to heart failure were defined as the *secondary endpoint*. In case of simultaneous cardiac events per patient, the worst event was selected (cardiac death > transplantation > aborted SCD due to appropriate ICD discharge > hospitalization due to heart failure). There was lack of follow-up data from 45 patients from the EuroCMR Registry.

### Statistical analysis

Continuous parameters were expressed as mean ± standard deviation for parametric and as median with interquartile range (IQR) for nonparametric variables. Normal distribution was assessed using Shapiro–Wilk test. To compare continuous variables between two groups, Student’s t-test and Mann Whitney U test were used as applicable. Categorical variables were expressed as counts and proportions and were compared using the Chi-square test. The Kaplan–Meier method was used for survival curves, which were compared by log-rank tests. Receiver operating characteristics analysis was used to define optimal cut-off values for the prediction of clinical endpoints. Univariate and multivariate Cox proportional hazards regression analyses were performed to calculate hazard ratios (HR) and 95% confidence intervals (CI). Collinearity was examined using a correlation matrix of correlation coefficients (r) for the observed parameters after the method of Pearson et al. for parametric and Spearman et al. for non-parametric parameters. Intra- and interobserver variabilities for GLS, GCS, GRS and LV torsion were assessed by repeated analysis of 20 randomly selected patients of the control group and 20 DCM patients. The intra- and interobserver variability was described using the intra-class correlation coefficient (ICC with 95% CI) with a two-way random model with absolute agreement. A p-value of < 0.05 was regarded as statistically significant. Dedicated statistical software MedCalc (v20.013, MedCalc software, Mariakerke, Belgium) was used for statistical analysis.

## Results

**Fig. 2:**
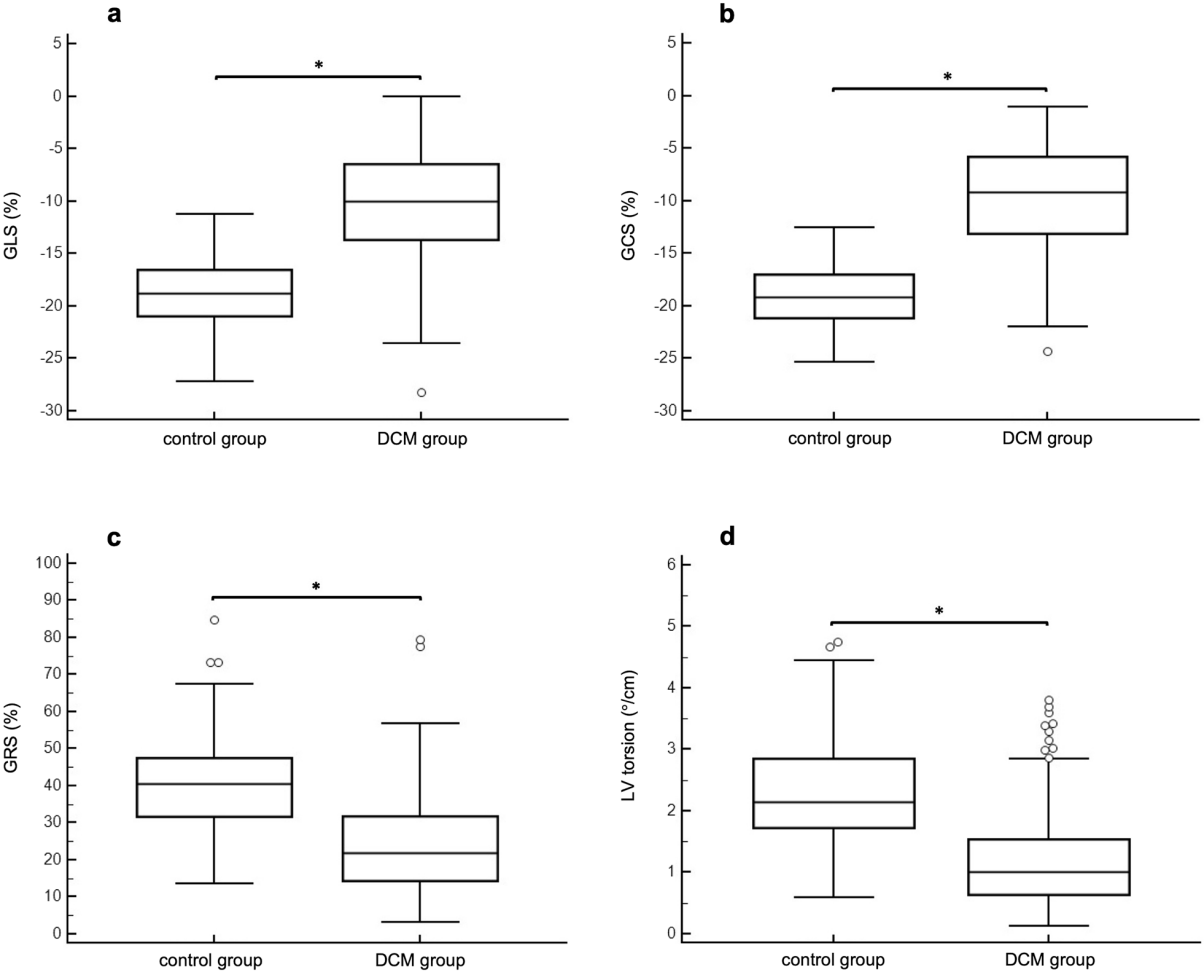
Comparison of strain parameters: healthy control group vs. dilated cardiomyopathy (DCM) group. Box-whisker-plots for the comparison of different strain parameters including LV torsion between the healthy control and DCM group. **a** global longitudinal strain (GLS), **b** global circumferential strain (GCS), **c** global radial strain, (GRS) **d** LV torsion; * p < 0.001

The final study population consisted of 350 subjects (259 male; 52.2 ± 15.2 years). There were no significant differences regarding age and gender between the DCM- and the control group (Table 1). Most patients had dyspnea in New York Heart Association (NYHA) class II and III (n = 260; 74.3%), they presented with various cardiovascular risk factors. Almost every patient was treated with an angiotensin converting enzyme (ACE) inhibitor or angiotensin receptor blocker (ARB; 346 patients, 98.9%). LVEF was significantly lower, whereas N-terminal pro-hormone brain natriuretic peptide (NT-pro BNP) was significantly higher in DCM patients compared to the healthy control group (Tables 1 and 2, all p < 0.001). LGE was present in 134 (38.3%) DCM patients. GLS, GCS, GRS and LV torsion were significantly impaired in the DCM cohort (Table 2, Fig. 2). Especially LVEF, GLS and GCS correlated strongly (Additional file 1: Table S1).

**Table 1:** Patient characteristics of the healthy—and dilated cardiomyopathy (DCM) group

	Healthy group (n = 70)	DCM group (n = 350)	p-value
Age [years]	51.9 ± 15.2	52.2 ± 15.2	0.87
Male gender [n]	46 (65.7%)	259 (73.9%)	0.16
Weight [kg]	75.9 ± 10.4	79.8 ± 15.7	< 0.01
Height [cm]	174.4 ± 8.9	175.8 ± 9.3	0.26
BMI [kg/m^2^]	24.9 ± 3.0	25.8 ± 4.3	< 0.05
NYHA class [n]			
I	70 (100.0%)	84 (24.0%)	< 0.001
II	0	152 (43.4%)	< 0.001
III	0	108 (30.9%)	< 0.001
IV	0	6 (1.7%)	< 0.001
Cardiovascular risk factors [n]			
Arterial hypertension	0	154 (44.0%)	< 0.001
Dyslipidemia	0	89 (25.4%)	< 0.001
Diabetes	0	42 (12.0%)	< 0.001
Smoker	12 (17.1%)	140 (40.0%)	< 0.001
Family history of CAD	0	77 (22.0%)	< 0.001
Obesity	4 (5.7%)	52 (14.9%)	< 0.001
Heart failure therapy			
ACE inhibitor/ARB	0	346 (98.9%)	< 0.001
ß-Blocker	0	300 (85.7%)	< 0.001
Aldosterone receptor antagonist	0	145 (41.4%)	< 0.001
Loop diuretic	0	169 (48.3%)	< 0.001
Heart rate [/min]	68.5 ± 10.1	71.4 ± 15.2	0.05
Atrial fibrillation [n]	0	55 (15.7%)	< 0.001
Laboratory findings			
Hemoglobine [g/dl]	14.5 (13.5–15.4)	14.3 (13.3–15.4)	0.14
Creatinine [mg/dl]	0.9 (0.7–1.0)	0.9 (0.8–1.1)	0.10
NT-pro BNP [ng/l]	39.0 (24.0–61.0)	373.0 (124.0–1797.0)^†^	< 0.001

*ACE* angiotensin converting enzyme, *ARB* angiotensin receptor blocker, *BMI* body mass index, *CAD* coronary artery disease, *NT-pro BNP* N-terminal pro-hormone brain natriuretic peptide, *NYHA* New York Heart Association

^†^NT-pro BNP values of 110 patients missing

**Table 2:** Comparison of CMR parameters including LV strain and rotational parameters between healthy—and DCM group

	Healthy group (n = 70)	DCM group (n = 350)	p-value
LVEF [%]	60.1 ± 5.1	36.4 ± 13.7	< 0.001
LVEDV [ml]	168.0 ± 38.2	248.9 ± 94.4	< 0.001
LV mass [g]	77.7 ± 20.9	147.3 ± 97.0	< 0.001
presence of LGE [n]	–	134 (38.3%)	–
GLS [%]	− 17.5 ± 3.8	− 9.9 ± 4.5	< 0.001
GCS [%]	− 19.1 ± 3.0	− 9.4 ± 4.9	< 0.001
GRS [%]	41.8 ± 14.6	23.5 ± 12.0	< 0.001
Apical rotation [°]	6.1 ± 3.0	2.0 ± 4.1	< 0.001
Basal rotation [°]	− 4.8 ± 4.0	− 2.5 ± 3.6	< 0.001
LV twist [°]	10.3 ± 3.7	5.5 ± 3.0	< 0.001
LV torsion [°/cm]	2.3 ± 0.9	1.2 ± 0.7	< 0.001
Normal directed rotation [n]	62 (88.6%)	191 (54.6%)	< 0.001
Reversed apical rotation [n]	1 (1.4%)	98 (28.0%)	< 0.001

*GCS* global circumferential strain, *GLS* global longitudinal strain, *GRS* global radial strain, *LVEDV* left ventricular end-diastolic volume, *LGE* late gadolinium enhancement, *LVEF* left ventricular ejection fraction

Patients from EuroCMR Registry had a significantly lower LVEF (EuroCMR: 28.7 ± 9.7% vs. Heidelberg: 37.5 ± 13.9%; p < 0.001), while age (EuroCMR: 55.5 ± 15.7 years vs. Heidelberg: 51.7 ± 15.1 years; p = 0.12) and gender (EuroCMR: 63.6% males vs. Heidelberg: 75.4% males; p = 0.10) were similar.

In the DCM subgroup with LGE (n = 134); GLS (− 10.6 ± 4.2% vs. − 8.7 ± 4.6%, p < 0.001), GCS (− 10.4 ± 4.4% vs. − 8.6 ± 5.1%, p < 0.001), GRS (25.5 ± 12.1% vs. 20.3 ± 11.3%, p < 0.001) as well as the LVEF (38.9 ± 13.0% vs. 32.3 ± 13.9%, p < 0.001) but not LV torsion (1.2 ± 0.7°/cm vs. 1.1 ± 0.6°/cm, p = 0.12) were significantly reduced compared to DCM patients *without* LGE.

In contrast to the healthy control group (p < 0.001), a considerable fraction of DCM patients showed a reversed direction of rotation (45.4%), predominantly affecting the LV apex (n = 98 patients; 28.0%). Instead of a counterclockwise apical rotation, what is regarded to be normal in systole, these patients showed a peak clockwise rotation of the apex resulting in a systolic rotation of apex and base into the same direction. Compared to DCM patients with normally directed rotation, the patients with reversed apical rotation had a significantly lower LVEF (38.5 ± 13.6% vs. 30.8 ± 12.6%; p < 0.001). Regarding LV strain and LV rotation, especially LV torsion was impaired (1.3 ± 0.7°/cm vs. 0.8 ± 0.6°/cm; p < 0.001). In the group of reversed apical rotation, a larger proportion of patients had a left bundle branch block (LBBB) compared to the group of patients with normally directed rotation (42.9% vs. 13.6%, p < 0.001). Consequently, QRS duration was significantly prolonged in patients with reversed apical rotation (125 ± 31 ms vs. 109 ± 23 ms; p < 0.001). Thereby, reversed rotation of the LV apex provided high specificity (specificity = 98.6%, sensitivity = 28.0%) for the diagnosis of DCM.

Some subjects of the control group showed a reversed rotation direction (n = 8, 11.4%), especially of the LV base. One subject had a reversed apical rotation (1.4%). Clinical, CMR or other LV strain parameters showed no significant differences compared to the subjects with normally directed rotation.

### Baseline follow-up data

Over a median follow-up period of 4.2 (2.0–5.6) years, the primary endpoint was observed in 59 patients (18.7%), the secondary endpoint in 73 patients (20.9%). In total, 84 events occurred: 25 patients experienced a cardiac death, 7 underwent heart transplantation, 28 had an aborted SCD by an appropriate ICD discharge and 24 patients were hospitalized for heart failure.

The patients, in whom the primary endpoint occurred, had a significantly impaired LVEF, GLS, GCS, GRS and LV torsion (all p < 0.05; Table 3).

**Table 3:** Comparison of DCM patients without and with a primary endpoint

	No event (n = 291)	Primary endpoint (n = 59)	p-value
Age [years]	51.6 ± 15.2	55.2 ± 15.3	0.10
Male gender [n]	214 (73.5%)	45 (76.3%)	0.66
Weight [kg]	80.5 ± 16.0	76.4 ± 14.0	0.07
Height [cm]	176.0 ± 8.9	174.8 ± 11.1	0.44
BMI [kg/m^2^]	25.9 ± 4.4	24.9 ± 3.4	< 0.05
Heart rate [1/min]	70.8 ± 15.2	74.4 ± 15.0	0.09
Atrial fibrillation [n]	46 (15.8%)	11 (18.6%)	0.58
NYHA class [n]			
I	72 (24.7%)	12 (20.3%)	0.50
II	133 (45.7%)	19 (32.2%)	0.08
III	80 (27.5%)	28 (47.5%)	< 0.01
IV	6 (2.1%)	0	–
Cardiovascular risk factors [n]			
Arterial hypertension	127 (43.6%)	27 (45.8%)	0.75
Dyslipidemia	76 (26.1%)	13 (22.0%)	0.51
Diabetes	31 (10.7%)	11 (18.6%)	0.14
Smoker	119 (40.9%)	21 (35.6%)	0.50
Family history of CAD	63 (21.6%)	14 (23.7%)	0.66
Obesity	47 (16.2%)	5 (8.5%)	0.08
Heart failure therapy [n]			
ACE inhibitor/ARB	287 (98.6%)	59 (100.0%)	0.57
ß-Blocker	245 (84.2%)	54 (91.5%)	0.11
Aldosterone receptor antagonist	112 (38.5%)	34 (57.6%)	< 0.01
Loop diuretic	128 (44.0%)	42 (71.2%)	< 0.001
Laboratory findings			
Creatinine [mg/dl]	0.9 (0.8–1.1)	1.1 (0.9–1.3)	0.45
NT-pro BNP [ng/l]^†^	275.0 (102.3–1229.8)	1831 (411.3–4976.3)	< 0.05
CMR parameters			
LVEF [%]	38.2 ± 13.2	27.1 ± 12.7	< 0.001
LVEDV [ml]	239 ± 81	298 ± 133	< 0.01
LV mass [g]	139 ± 50	189 ± 205	0.07
Presence of LGE [n]	97 (33.3%)	37 (62.7%)	< 0.001
GLS [%]	− 10.3 ± 4.5	− 7.5 ± 3.7	< 0.001
GCS [%]	− 10.0 ± 4.9	− 6.6 ± 3.6	< 0.001
GRS [%]	24.7 ± 12.2	17.7 ± 9.4	< 0.001
Apical rotation [°]	1.9 ± 4.2	2.3 ± 3.9	0.36
Basal rotation [°]	− 2.5 ± 3.7	− 2.3 ± 3.0	0.84
LV twist [°]	5.6 ± 3.1	5.0 ± 2.5	0.11
LV torsion [°/cm]	1.2 ± 0.7	1.0 ± 0.6	< 0.05
Normal directed rotation [n]	159 (54.6%)	32 (54.2%)	0.98
Reversed apical rotation [n]	89 (27.1%)	15 (25.4%)	0.84

^†^NT-pro BNP values of 110 patients missing

In the subgroup of DCM patients with an LVEF ≤ 35% (n = 160), the primary endpoint was observed in 44 patients (27.5%). In these 44 patients a significantly lower GLS (− 5.9 ± 2.7% vs. − 7.4 ± 3.4%, p < 0.01) and GCS (− 5.0 ± 2.0% vs. − 6.2 ± 2.8%, p < 0.01) was observed.

In contrast, only 15 (7.9%) primary endpoint events were observed in the patient group with an LVEF > 35% (n = 190). GLS (− 12.4 ± 3.9% vs. − 11.3 ± 3.6%, p = 0.14), GCS (− 12.9 ± 3.8% vs. − 11.3 ± 2.8%, p = 0.12), GRS (29.5 ± 11.8%, vs. 25.4 ± 9.6%, p = 0.19) and the LV torsion (1.4 ± 0.7°/cm vs. 1.3 ± 0.7°/cm, p = 0.59) were lower in these 15 patients—but only as a trend—compared to the other patients with a LVEF > 35%.

### Uni- and multivariate analysis

Several clinical (age, NYHA class), laboratory (log transformed NT-pro BNP), standard CMR (LVEF, LV end-diastolic volume (LVEDV), presence of LGE) and strain parameters (GLS, GCS, GRS, LV torsion) were associated with the primary and the secondary endpoint in univariate analysis (Table 4 and Additional file 1: Table S2).

**Table 4:** Univariate survival analysis of the primary endpoint (Cox proportional-hazards regression)

	Primary endpoint			
	HR	95% CI	p-value	χ^2^
Age [years]	1.02	1.00–1.04	< 0.05	4.17
NYHA	1.49	1.04–2.14	< 0.05	4.85
LVEF [%]	0.94	0.93–0.96	< 0.001	35.85
LVEDV [ml]	1.01	1.00–1.01	< 0.001	17.14
lnNT-pro BNP [ng/l]	1.52	1.32–1.75	< 0.001	32.15
LGE [n]	3.05	1.79–5.22	< 0.001	17.58
GLS [%]	1.22	1.14–1.31	< 0.001	36.81
GCS [%]	1.23	1.14–1.32	< 0.001	40.06
GRS [%]	0.94	0.91–0.97	< 0.001	21.41
LV twist [°]	0.92	0.84–1.02	0.09	2.89
LV torsion [°/cm]	0.63	0.40–0.97	< 0.05	4.90
Reversed apical rotation [n]	1.08	0.61–1.90	0.79	0.07

NT-pro BNP was log transformed (lnNT-pro BNP)

Based on the univariate analysis of LV strains, a multivariate Cox regression model including seven different parameters was assessed (Table 5 and Additional file 1: Table S3). Besides standard CMR parameters used for risk stratification in DCM (LVEF, LVEDV and the presence of LGE), the strain parameters (GLS, GCS, GRS and LV torsion) were included. In this multivariate, backwards regression model only the presence of LGE for the primary endpoint (HR 2.16, 95% CI 1.24–3.77, p < 0.05) and GLS for primary (HR 1.13, 95% CI 1.01–1.26, p < 0.05) and secondary endpoint (HR 1.11, 95% CI 1.01–1.23, p < 0.05) remained as independent predictors.

**Table 5:** Multivariate survival analysis of the primary endpoint (Cox proportional-hazards regression)

	Primary endpoint		
	HR	95% CI	p-value
LVEF [%]	1.00	0.95–1.05	0.90
LVEDV [ml]	1.00	1.00–1.01	0.50
LGE [n]	2.16	1.24–3.77	< 0.05
GLS [%]	1.13	1.01–1.26	< 0.05
GCS [%]	1.07	0.92–1.24	0.38
GRS [%]	0.99	0.96–1.03	0.71
LV torsion [°/cm]	1.26	0.74–2.13	0.40

**Fig. 3:**
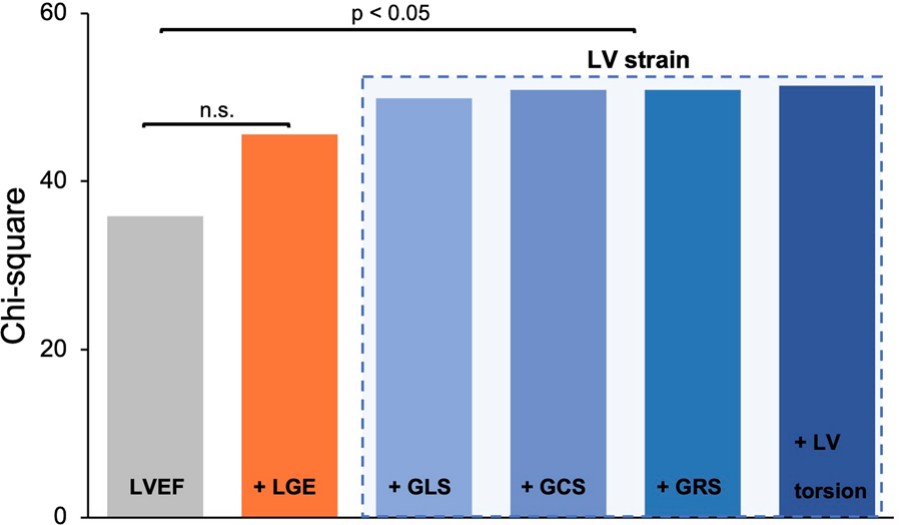
Cox proportional-hazards model of the additional value of LV strain parameters. Strain parameters and late gadolinium enhancement (LGE) improve risk stratification significantly compared to LV ejection fraction (LVEF). p < 0.05

Furthermore, series of Cox proportional-hazards models with stepwise regression were created to evaluate the incremental value of strain imaging using CMR FT (Fig. 3). As demonstrated, the different LV strain parameters, particularly GLS as well as LGE provided incremental information compared to standard risk stratification with LVEF (p < 0.05).

### Survival analysis

**Fig. 4:**
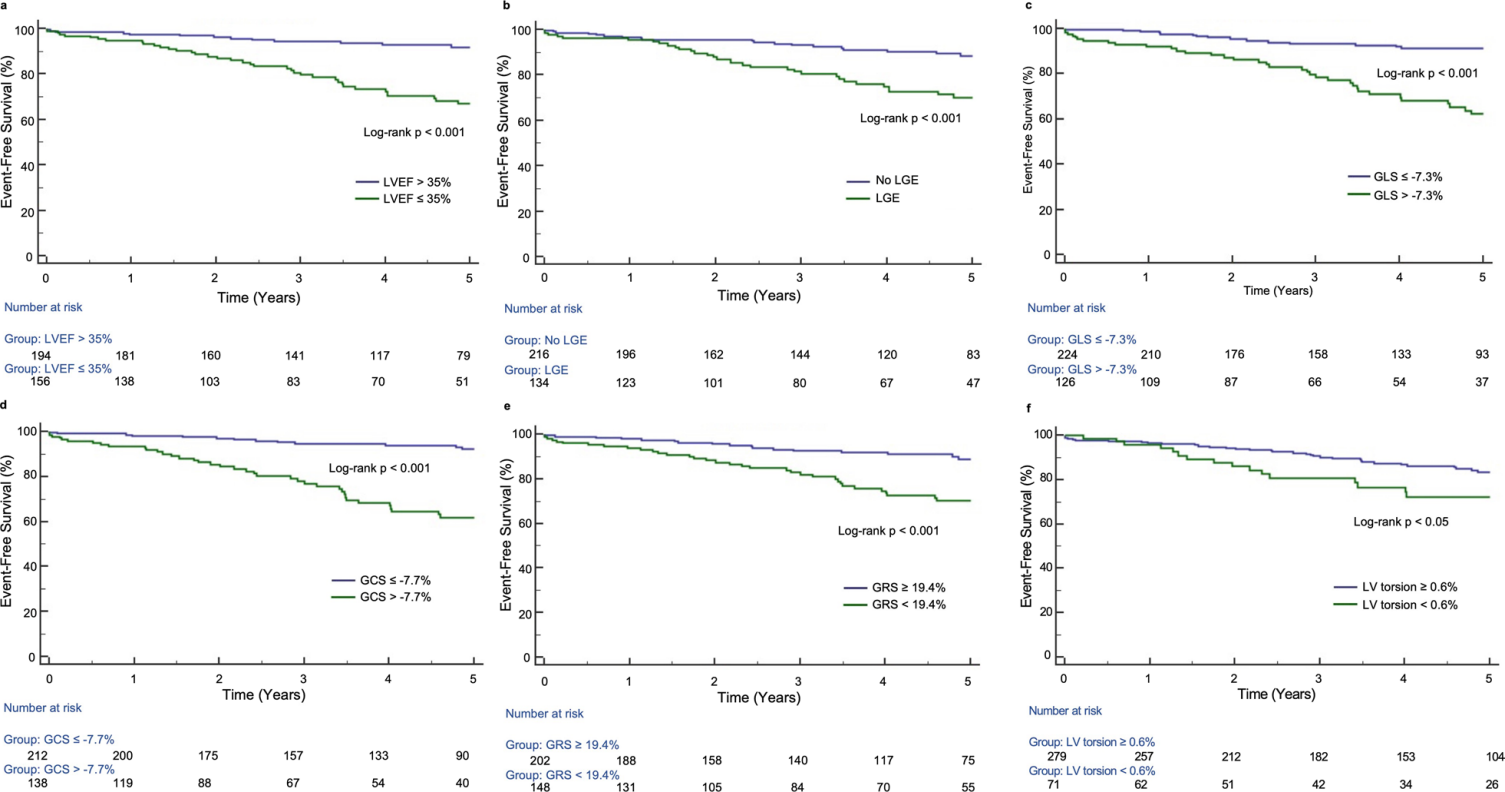
Kaplan–Meier survival analyses for the prediction of primary endpoint. Kaplan–Meier curves for LVEF (**a**) with cut-off value of ≤ 35%, the presence of LGE (**b**), GLS (**c**), GCS (**d**), GRS (**e**) and LV torsion (**f**)

For the Kaplan–Meier survival analyses, LV torsion was used as the only rotational parameter due to its higher prognostic value as described above (Table 4). Cut-off values were determined using ROC curve analyses of the primary endpoint: -7.3% for GLS (AUC = 0.72, p < 0.001), -7.7% for GCS (AUC = 0.73, p < 0.001), 19.4% for GRS (AUC = 0.68, p < 0.001) and 0.6°/cm for the LV torsion (AUC = 0.58, p < 0.05). Consequently, in Kaplan–Meier analysis, patients with a GLS > − 7.3%, a GCS > − 7.7%, a GRS < 19.4% (all log-rank p < 0.001) and a LV torsion < 0.6°/cm (log-rank p < 0.05) had a significantly higher rate of cardiac events (Fig. 4). LVEF ≤ 35% as well as the presence of LGE were also significant predictors of the primary endpoint (all log-rank p < 0.001; Fig. 4).

**Fig. 5:**
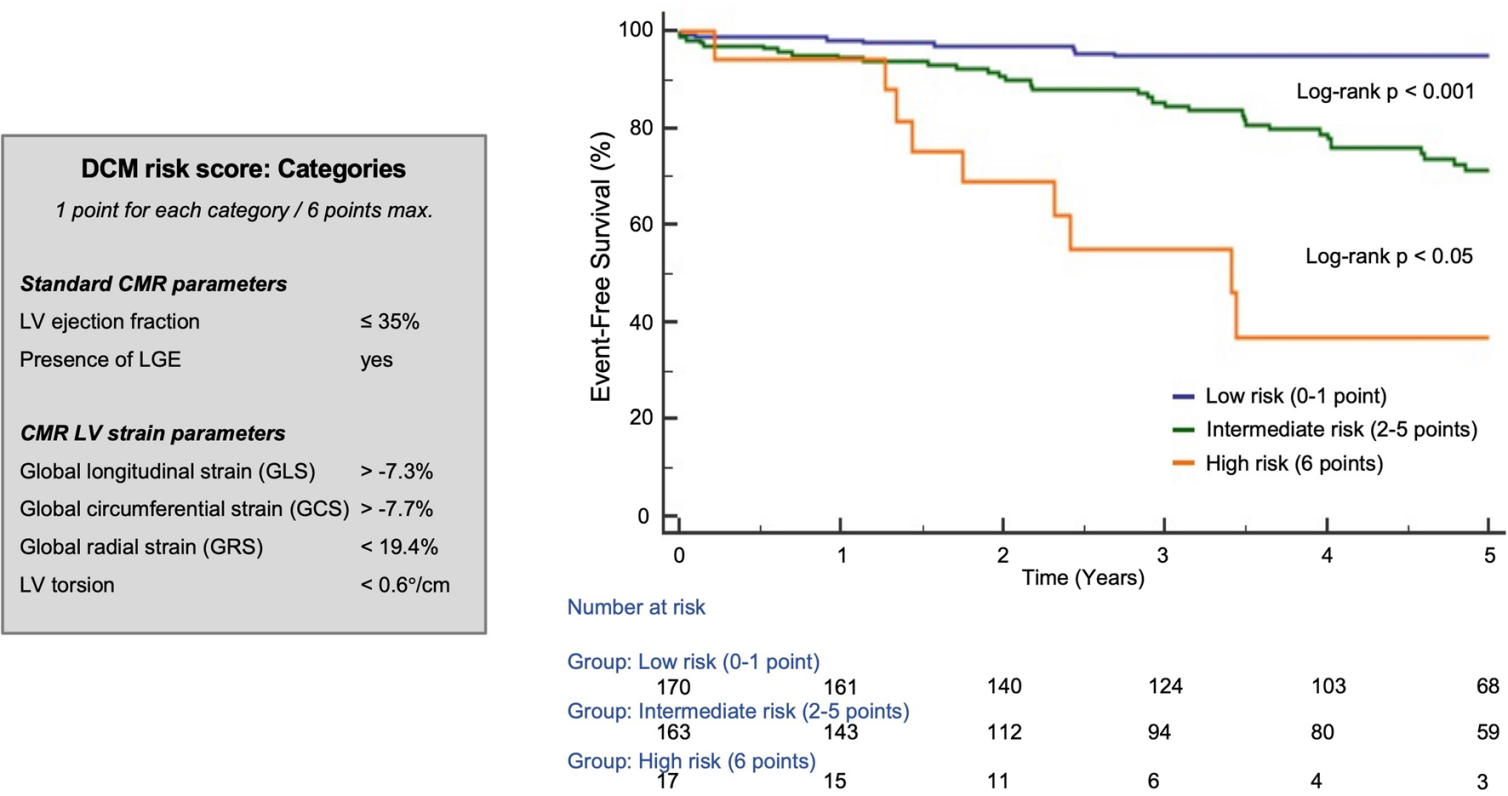
Kaplan–Meier analysis of a CMR risk score in DCM patients. CMR risk score is based on different CMR (LVEF, LGE) and strain parameters (GLS, GCS, GRS, LV torsion) for the prediction of the primary endpoint (cut-off values as mentioned on the left): low risk (0–1p), intermediate risk (2–5p) and high risk (6p)

Based on different CMR and strain parameters, a DCM risk score was built, including LVEF ≤ 35% and the presence of LGE as standard CMR parameters. The addition of dichotomized LVEDV led to a worsening of the prognostic power, hence LVEDV was not part of DCM risk score. LV strains and LV torsion completed the six different categories of the scoring model. One point was added for each category worse than the above-mentioned cut-off values. Three main groups were created: a low risk- (0–1 point), an intermediate risk- (2–5 points) and a high-risk group (6 points). In Kaplan–Meier analysis, each group differed significantly from each other (primary and secondary endpoint; p < 0.05; Fig. 5 and Additional file 2: Fig. S1). Even a simplified risk score, only consisting of LVEF ≤ 35%, the presence of LGE and GLS > -7.3% allows for excellent differentiation between patients at low (0–2 points) and high risk (3 points) for a negative clinical outcome (log-rank p < 0.001; Additional file 2: Fig. S2).

### Observer variability

Quantification of the different LV strain and LV rotational parameters (GLS, GCS, GRS, LV torsion) using FT in CMR featured a good reproducibility in healthy subjects as well as DCM patients (Additional file 1: Table S4a and S4b). The ICC for the intraobserver variability in healthy subjects ranged between 0.93 (95% CI: 0.72–0.98) for LV torsion and 0.98 (95% CI: 0.93–0.99) for GLS, the ICC for the interobserver variability between 0.85 (95% CI: 0.43–0.96) for LV torsion and 0.96 (95% CI: 0.84–0.99) for GLS. In DCM patients, intra- and interobserver reproducibility was lower but still good: ICC for intraobserver variability ranged between 0.91 (95% CI: 0.65–0.98) for LV torsion and 0.98 (95% CI: 0.80–0.99) for GLS, for interobserver variability between 0.84 (95% CI: 0.40–0.96) for LV torsion and 0.96 (95% CI: 0.77–0.98) for GLS.

## Discussion

In this study, including subjects from five different European CMR centers, the alterations of myocardial mechanics in DCM patients were assessed and compared to a control group of proven healthy subjects. To our knowledge, this is the first study assessing GLS, GCS, GRS and LV torsion in a combined approach providing prognostic values for strain as well as LV rotational parameters.

The main findings of our study were: (1) strain and rotational parameters could be assessed reliably using CMR FT with high intra- and interobserver reproducibility. (2) GLS, GCS, GRS as well as LV torsion were significantly impaired in patients with DCM. (3) The analyzed parameters offered a significant prognostic value surpassing standard CMR parameters for risk stratification.

### Prognostic value of LV strain parameters

The timely diagnosis of DCM and recognition of myocardial dysfunction is important for the initiation of therapy, risk stratification and to reduce mortality [24]. Currently, global LVEF is used for the quantification of LV systolic function and is commonly used to guide the initiation of medical heart failure therapy. It is also employed as a prognostic marker and as a parameter for ICD and resynchronization therapy eligibility [3].

In accordance with previous studies on myocardial mechanics in DCM, a reduction of all strain parameters including LV torsion was observed [25, 26]. LV strain assessed with CMR FT provided incremental prognostic value and should be considered as clinical routine diagnostics in patients with DCM. Thereby, all strain parameters were significantly associated with the primary and secondary endpoint in univariate analysis. In a multivariate model including standard CMR parameters, GLS remained as the only independent predictor of both clinical endpoints. This finding has been observed in various other cardiac pathologies including acute myocardial infarction, after heart transplantation, myocarditis and in patients with preserved LVEF suggesting that GLS may represent a suitable parameter for global cardiac function assessment and prognostication [27–30]. The assessment of myocardial strains has therefore shown to improve risk stratification surpassing classical clinical, echocardiographic or CMR-derived risk features [1, 15, 31].

In DCM patients, a more precise prediction of clinical outcome using GLS was reported by several investigators—some of these included patients from our study population [15, 17, 20, 32]. This observation was confirmed in our large cohort of DCM patients in which we further examined LV rotation. LV rotational parameters were altered in DCM patients, however, they were no independent risk predictors due to the strong predictive value of LGE and GLS.

A risk score including six categorical variables of standard CMR and strain parameters allowed for further risk stratification in DCM patients.

Remarkably, LGE was an independent predictor of the primary endpoint but not of the secondary endpoint, which additionally includes hospitalization due to heart failure. Of note, GLS was independently associated with both endpoints. Thus, an impaired GLS may represent a better indicator of a worse outcome. Regarding LGE, myocardial fibrosis may not only be a result of an advanced stage of DCM but also increase the risk of adverse events as ventricular arrhythmias.

A comprehensive approach assessing both functional parameters including LV strain and tissue composition, as done in this study, may be beneficial in clinical routine.

In this respect, we were able to prove the additional prognostic value of LV strain in advanced disease stages even in our subgroup analysis: in patients with a LVEF ≤ 35% (n = 160), GLS and GCS were still significant predictors of the primary endpoint. Since the identification of patients eligible for an ICD remains challenging, especially in non-ischemic cardiomyopathies as shown by the DANISH study, the addition of strain parameters may improve the risk stratification [33].

### Prognostic value of LV rotational parameters

LV rotation prognostic data are scarce and to our knowledge, we provide the first prognostic data of CMR LV torsion in DCM. The direction of apical rotation may be a hallmark of DCM as many patients showed a reversed apical rotation. The pathogenesis of reversed apical rotation is unclear, though in our study population these patients had significantly more often a LBBB than patients with normal directed rotation. LV rotational mechanics might be influenced by a changed propagation of cardiac excitation: former studies reported of a delayed activation of apical myocardium and an ‘U-shaped’ activation from right to left of at first anterior epicardium followed by septum and then of the lateral wall in patients with LBBB [34, 35]. It might be hypothesized that this changed, delayed excitation results in a reversed apical rotation.

In patients with DCM a reversed apical rotation has been reported, however its prognostic potential was previously unknown [25, 26]. In an echocardiographic study with 50 DCM patients, Popescu et al. observed a lower LVEF in 26 patients with a reversed apical rotation [25]. They proposed, that a reversed apical rotation in DCM patients could be associated with a more severe disease stage. Although LVEF was significantly reduced in patients with reversed apical rotation, we did not observe a significant influence of the direction of rotation on prognosis. Regarding the LV rotational parameters, only LV torsion was a predictor of clinical outcome.

In another study, Rady et al. previously showed an incremental value of LV torsion assessed with speckle-tracking echocardiography compared to right ventricular function and peak VO2 for the prediction of cardiac events in 91 DCM patients and a median follow-up of 272 days [36].

As we were able to prove in this study, LV torsion as a rotational parameter had a significant prognostic value, which, however, is lower than other strain parameters like GLS or GCS. Thereby, GLS and GCS represent the strongest strain parameters with regard to their prognostic value.

### Strain imaging for the evaluation of myocardial mechanics

CMR FT provides quick and reliable global strain and rotational values with good intra- and inter-observer variability [6, 8, 37, 38]. Additional information about myocardial mechanics beyond LVEF can be obtained without the need of additional, dedicated CMR sequences. While echocardiography allows for an easy and straightforward assessment of LV morphology and function inclusive of strain measurements using speckle-tracking, CMR offers a more comprehensive approach for risk stratification. In CMR, standard LV parameters as LVEF not only show a better reproducibility but also a higher prognostic value than in echocardiography [39]. Risk stratification can be further enhanced by strain measurements, which are not limited by the patient’s acoustic window. In addition, CMR tissue characterization as LGE adds valuable prognostic information in ischemic and non-ischemic cardiomyopathies [40, 41]. Hence, CMR not only gains importance in clinical practice and current guidelines but is also assessed with regard to therapy optimization in prospective trials [42].

The application of fully automated software approaches for strain analysis could accelerate this development even further [43].

### Limitations

CMR FT strain analysis has been previously validated, but there are no data on the agreement of LV rotational measurements between CMR FT and CMR tagging. There are only feasibility studies using CMR FT to assess LV rotation [10, 44]. In general, measurements of cardiac rotation are less robust than longitudinal parameters [10]. Therefore, the tracking was meticulously checked by an experienced reader in this study. Furthermore, we favored global over segmental values for analysis and repeated all strain measurements three times. As FT is dependent on good image quality and an exact endocardial border definition, we had to exclude 15 patients due to an insufficient tracking quality.

As part of the study population was drawn from the EuroCMR Registry, the quality of clinical and CMR-derived data of these patients were dependent on the respective institution. Due to the lack of follow-up data, 45 patients of EuroCMR Registry had to be excluded resulting in a predominance of a single center.

Because NT-pro BNP values were only available in 68.5% of the DCM patients, its prognostic value is possibly underestimated. As we showed a strong correlation between LVEF, GLS and GCS, there is a certain collinearity among the different parameters.

A priori power analysis regarding the prognostic value of LV torsion was not possible due to missing prognostic data. As rotational parameters were significant predictors in the univariate but not the multivariate analyses, one could speculate that they might have become statistically significant in a larger study population. However, the clinical relevance of such small effects would be questionable.

## Conclusions

In patients with DCM, global LV strain parameters showed an additive prognostic value. Especially GLS surpassed standard clinical and CMR-derived parameters in the risk stratification of DCM patients. GLS and GCS add further prognostic information in DCM patients even in subjects with a LVEF ≤ 35%. The DCM risk score as a simple scoring system including standard CMR- and strain parameters allows for an exact risk stratification in DCM patients. With its additional prognostic value, an easy-to-use, fast and reproducible assessment of strain and rotational parameters, CMR FT may aid in risk stratification of DCM patients—and may find its way to routine clinical application. Larger, prospective multi-centre trials, using a comparable global strain approach, are needed to confirm our findings.

## Supplementary Information

**Additional file 1: Table S1.** Correlation matrix for correlation coefficients (r) of common CMR and strain parameters including LV torsion. LVEF: left ventricular ejection fraction; GLS: global longitudinal strain; GCS: global circumferential strain; GRS: global radial strain; LV torsion: left ventricular torsion; LVEDV: left ventricular end-diastolic volume. **Table S2.** Univariate survival analysis of the secondary endpoint (Cox proportional-hazards regression). HR: hazard ratio; CI: confidence interval; LVEF: left ventricular ejection fraction; LVEDV: left ventricular end-diastolic volume; NT-pro BNP: N-terminal pro-hormone brain natriuretic peptide; LGE: late gadolinium enhancement; GLS: global longitudinal strain; GCS: global circumferential strain; GRS: global radial strain. **Table S3.** Multivariate survival analysis of the secondary endpoint (Cox proportional-hazards regression). HR: hazard ratio; CI: confidence interval; LVEF: left ventricular ejection fraction; LVEDV: left ventricular end-diastolic volume; LGE: late gadolinium enhancement; GLS: global longitudinal strain; GCS: global circumferential strain; GRS: global radial strain. **Table S4a.** Intra- and Interobserver variability of the strain parameters in healthy individuals. ICC: intra-class correlation coefficient; CI: confidence interval; GLS: global longitudinal strain; GCS: global circumferential strain; GRS: global radial strain; LV: left ventricular. **Table S4b.** Intra- and Interobserver variability of the strain parameters in DCM patients. ICC: intra-class correlation coefficient; CI: confidence interval; GLS: global longitudinal strain; GCS: global circumferential strain; GRS: global radial strain; LV: left ventricular.

**Additional file 2: Figure S1.** Kaplan–Meier analysis of a CMR risk score for the prediction of the secondary endpoint. CMR risk score is based on different CMR (LVEF, LGE) and strain parameters for the prediction of the secondary endpoint in DCM patients. Cut-off values as mentioned in Fig. 4: low risk (0-1p), intermediate risk (2-5p) and high risk (6p). CMR: cardiac magnetic resonance imaging; DCM: dilated cardiomyopathy; GLS: global longitudinal strain; GCS: global circumferential strain; GRS: global radial strain; LVEF: left ventricular ejection fraction; LGE: late gadolinium enhancement. **Figure S2**. Kaplan–Meier analysis of a simplified CMR risk score in DCM patients. The simplified CMR risk score is based on LVEF, LGE and GLS as the single strain parameter for the prediction of the primary endpoint (cut-off values as mentioned in Fig. 4 of the manuscript): low risk (0-2p) and high risk (3p). CMR: cardiac magnetic resonance imaging; DCM: dilated cardiomyopathy; LGE: late gadolinium enhancement; LVEF: left ventricular ejection fraction; GLS: global longitudinal strain.

## Data Availability

The datasets used and/or analyzed during the current study are available from the corresponding author on reasonable request.

## Abbreviations

ACE: Angiotensin converting enzyme
ARB: Angiotensin receptor blocker
ATP: Anti-tachycardia pacing
AUC: Area under the curve
bSSFP: Balanced steady-state free precession
CI: Confidence interval
CMR: Cardiovascular magnetic resonance
DCM: Dilated cardiomyopathy
ECG: Electrocardiogram
FT: Feature tracking
GCS: Global circumferential strain
GLS: Global longitudinal strain
GRS: Global radial strain
HR: Hazard ratio
ICC: Intraclass correlation coefficient
ICD: Implanted cardioverter defibrillator
IQR: Interquartile range
LBBB: Left bundle branch block
LGE: Late gadolinium enhancement
LV: Left ventricle/left ventricular
LVEDD: Left ventricular end-diastolic diameter
LVEDV: Left ventricular end-diastolic volume
LVEF: Left ventricular ejection fraction
NT-pro BNP: N-terminal pro-hormone brain natriuretic peptide
NYHA: New York Heart Association
RV: Right ventricle/right ventricular
SCD: Sudden cardiac death
SCMR: Society for Cardiovascular Magnetic Resonance
