# Supplementary material for: Myocardial mechanics in dilated cardiomyopathy: prognostic value of left ventricular torsion and strain
Source: J Cardiovasc Magn Reson. 2021 Dec 2;23:136. doi: 10.1186/s12968-021-00829-x (PMC8638178; doi:10.1186/s12968-021-00829-x)
Supplement: Supplementary file 1 — Additional file 1: Table S1. Correlation matrix for correlation coefficients (r) of common CMR and strain parameters including LV torsion. LVEF: left ventricular ejection fraction; GLS: global longitudinal strain; GCS: global circumferential strain; GRS: global radial strain; LV torsion: left ventricular torsion; LVEDV: left ventricular end-diastolic volume. Table S2. Univariate survival analysis of the secondary endpoint (Cox proportional-hazards regression). HR: hazard ratio; CI: confidence interval; LVEF: left ventricular ejection fraction; LVEDV: left ventricular end-diastolic volume; NT-pro BNP: N-terminal pro-hormone brain natriuretic peptide; LGE: late gadolinium enhancement; GLS: global longitudinal strain; GCS: global circumferential strain; GRS: global radial strain. Table S3. Multivariate survival analysis of the secondary endpoint (Cox proportional-hazards regression). HR: hazard ratio; CI: confidence interval; LVEF: left ventricular ejection fraction; LVEDV: left ventricular end-diastolic volume; LGE: late gadolinium enhancement; GLS: global longitudinal strain; GCS: global circumferential strain; GRS: global radial strain. Table S4a. Intra- and Interobserver variability of the strain parameters in healthy individuals. ICC: intra-class correlation coefficient; CI: confidence interval; GLS: global longitudinal strain; GCS: global circumferential strain; GRS: global radial strain; LV: left ventricular. Table S4b. Intra- and Interobserver variability of the strain parameters in DCM patients. ICC: intra-class correlation coefficient; CI: confidence interval; GLS: global longitudinal strain; GCS: global circumferential strain; GRS: global radial strain; LV: left ventricular. [file 12968_2021_829_MOESM1_ESM.docx]

**Table 1.** **Correlation matrix for correlation coefficients (r) of common CMR and strain parameters including LV torsion.**

|  | **LVEF** | **GLS** | **GCS** | **GRS** | **LV torsion** | **LVEDV** |
| --- | --- | --- | --- | --- | --- | --- |
| **LVEF** |  | -0.77 | -0.83 | 0.67 | 0.54 | -0.69 |
| **GLS** | -0.77 |  | 0.78 | -0.57 | -0.51 | 0.49 |
| **GCS** | -0.83 | 0.78 |  | -0.66 | -0.54 | 0.61 |
| **GRS** | 0.67 | -0.57 | -0.66 |  | 0.49 | -0.45 |
| **LV torsion** | 0.54 | -0.51 | -0.54 | 0.49 |  | -0.43 |
| **LVEDV** | -0.69 | 0.49 | 0.61 | -0.45 | -0.43 |  |

GCS: global circumferential strain; GLS: global longitudinal strain; GRS: global radial strain; LV: left ventricular; LVEDV: left ventricular end-diastolic volume; LVEF: left ventricular ejection fraction

**Table 2. Univariate survival analysis of the secondary endpoint (Cox proportional-hazards regression).**

|  | **secondary endpoint** | | | | |
| --- | --- | --- | --- | --- | --- |
|  | **HR** | **95% CI** | **p-value** | **χ²** |  |
| **Age [years]** | 1.02 | 1.00-1.04 | <0.01 | 6.72 |  |
| **NYHA** | 1.49 | 1.08-2.06 | <0.05 | 6.14 |  |
| **LVEF [%]** | 0.95 | 0.93-0.96 | <0.001 | 38.12 |  |
| **LVEDV [ml]** | 1.00 | 1.00-1.01 | <0.001 | 15.86 |  |
| **lnNT-pro BNP [ng/l]** | 1.48 | 1.31-1.68 | <0.001 | 36.39 |  |
| **LGE [n]** | 2.02 | 1.27-3.21 | <0.01 | 8.77 |  |
| **GLS [%]** | 1.19 | 1.12-1.27 | <0.001 | 37.56 |  |
| **GCS [%]** | 1.20 | 1.13-1.27 | <0.001 | 39.41 |  |
| **GRS [%]** | 0.95 | 0.93-0.97 | <0.001 | 19.30 |  |
| **LV twist [°]** | 0.98 | 0.91-1.06 | 0.61 | 0.26 |  |
| **LV torsion [°/cm]** | 0.72 | 0.49-0.99 | <0.05 | 3.21 |  |
| **Reversed apical rotation [n]** | 0.90 | 0.54-1.53 | 0.71 | 0.14 |  |

CI: confidence interval; HR: hazard ratio; LGE: late gadolinium enhancement; NT-pro BNP: N-terminal pro-hormone brain natriuretic peptide;

**Table 3.** **Multivariate survival analysis of the secondary endpoint (Cox proportional-hazards regression).**

|  | **secondary endpoint** | | | |  |
| --- | --- | --- | --- | --- | --- |
|  | **HR** | **95% CI** | **p-value** |  | |
| **LVEF [%]** | 0.98 | 0.95-1.02 | 0.43 |  | |
| **LVEDV [ml]** | 1.00 | 1.00-1.01 | 0.67 |  | |
| **LGE [n]** | 1.37 | 0.84-2.23 | 0.20 |  | |
| **GLS [%]** | 1.11 | 1.01-1.23 | <0.05 |  | |
| **GCS [%]** | 1.05 | 0.93-1.18 | 0.48 |  | |
| **GRS [%]** | 0.99 | 0.96-1.02 | 0.61 |  | |
| **LV torsion [°/cm]** | 1.39 | 0.88-2.19 | 0.16 |  | |

**Table 4a.** **Intra- and Interobserver variability of the strain parameters in healthy individuals.**

|  | **Intraobserver variability** | | **Interobserver variability** | |
| --- | --- | --- | --- | --- |
|  | ICC (95% CI) | Bias (95% CI) | ICC (95% CI) | Bias (95% CI) |
| **GLS** | 0.98 (0.93-0.99) | -0.30 (-1.23-0.64) | 0.96 (0.84-0.99) | -0.34 (-1.57-0.89) |
| **GCS** | 0.97 (0.89-0.99) | -0.58 (-1.36-0.20) | 0.92 (0.70-0.98) | -0.57 (-1.75-0.60) |
| **GRS** | 0.96 (0.86-0.99) | -1.06 (-4.39-2.26) | 0.96 (0.85-0.99) | 0.16 (-3.12-3.43) |
| **LV torsion** | 0.93 (0.72-0.98) | -0.03 (-0.18-0.12) | 0.85 (0.43-0.96) | 0.08 (-0.17-0.32) |

ICC: intra-class correlation coefficient; CI: confidence interval; GLS: global longitudinal strain; GCS: global circumferential strain; GRS: global radial strain; LV: left ventricular.

**Table 4b.** **Intra- and Interobserver variability of the strain parameters in DCM patients.**

|  | **Intraobserver variability** | | **Interobserver variability** | |
| --- | --- | --- | --- | --- |
|  | ICC (95% CI) | Bias (95% CI) | ICC (95% CI) | Bias (95% CI) |
| **GLS** | 0.98 (0.80-0.99) | -0.84 (-1.61- -0.06) | 0.96 (0.77-0.98) | -0.79 (-2.25-0.68) |
| **GCS** | 0.93 (0.72-0.98) | -0.21 (-1.67-1.25) | 0.91 (0.64-0.98) | -0.26 (-1.87-1.34) |
| **GRS** | 0.94 (0.77-0.99) | -0.79 (-4.69-3.11) | 0.93 (0.64-0.98) | 3.17 (0.13-6.20) |
| **LV torsion** | 0.91 (0.65-0.98) | -0.08 (-0.26-0.09) | 0.84 (0.40-0.96) | -0.09 (-0.32-0.14) |

ICC: intra-class correlation coefficient
