# Supplementary material for: Myocardial mechanics in dilated cardiomyopathy: prognostic value of left ventricular torsion and strain
Source: J Cardiovasc Magn Reson. 2021 Dec 2;23:136. doi: 10.1186/s12968-021-00829-x (PMC8638178; doi:10.1186/s12968-021-00829-x)
Supplement: Supplementary file 2 — Additional file 2: Figure S1. Kaplan–Meier analysis of a CMR risk score for the prediction of the secondary endpoint. CMR risk score is based on different CMR (LVEF, LGE) and strain parameters for the prediction of the secondary endpoint in DCM patients. Cut-off values as mentioned in Fig. 4: low risk (0-1p), intermediate risk (2-5p) and high risk (6p). CMR: cardiac magnetic resonance imaging; DCM: dilated cardiomyopathy; GLS: global longitudinal strain; GCS: global circumferential strain; GRS: global radial strain; LVEF: left ventricular ejection fraction; LGE: late gadolinium enhancement. Figure S2. Kaplan–Meier analysis of a simplified CMR risk score in DCM patients. The simplified CMR risk score is based on LVEF, LGE and GLS as the single strain parameter for the prediction of the primary endpoint (cut-off values as mentioned in Fig. 4 of the manuscript): low risk (0-2p) and high risk (3p). CMR: cardiac magnetic resonance imaging; DCM: dilated cardiomyopathy; LGE: late gadolinium enhancement; LVEF: left ventricular ejection fraction; GLS: global longitudinal strain. [file 12968_2021_829_MOESM2_ESM.docx]

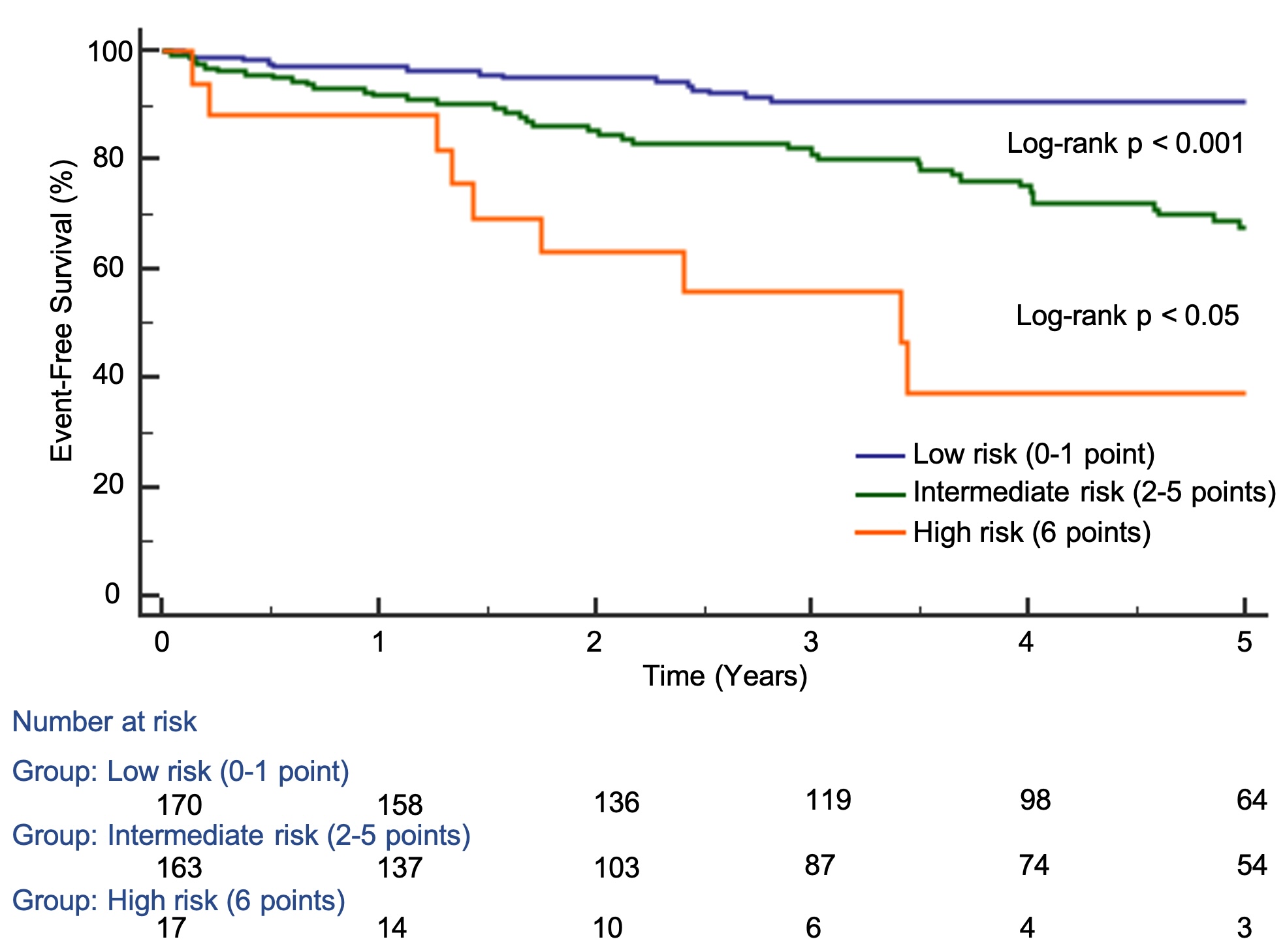


**Figure 1. Kaplan-Meier analysis of a CMR risk score** **for the prediction of the secondary endpoint**. CMR risk score is based on different CMR (LVEF, LGE) and strain parameters for the prediction of the secondary endpoint. Cut-off values as mentioned in Figure 4: low risk (0-1p), intermediate risk (2-5p) and high risk (6p).

CMR: cardiac magnetic resonance imaging; DCM: dilated cardiomyopathy; LVEF: left ventricular ejection fraction; LGE: late gadolinium enhancement; GLS: global longitudinal strain; GCS: global circumferential strain; GRS: global radial strain; LV torsion: left ventricular torsion.


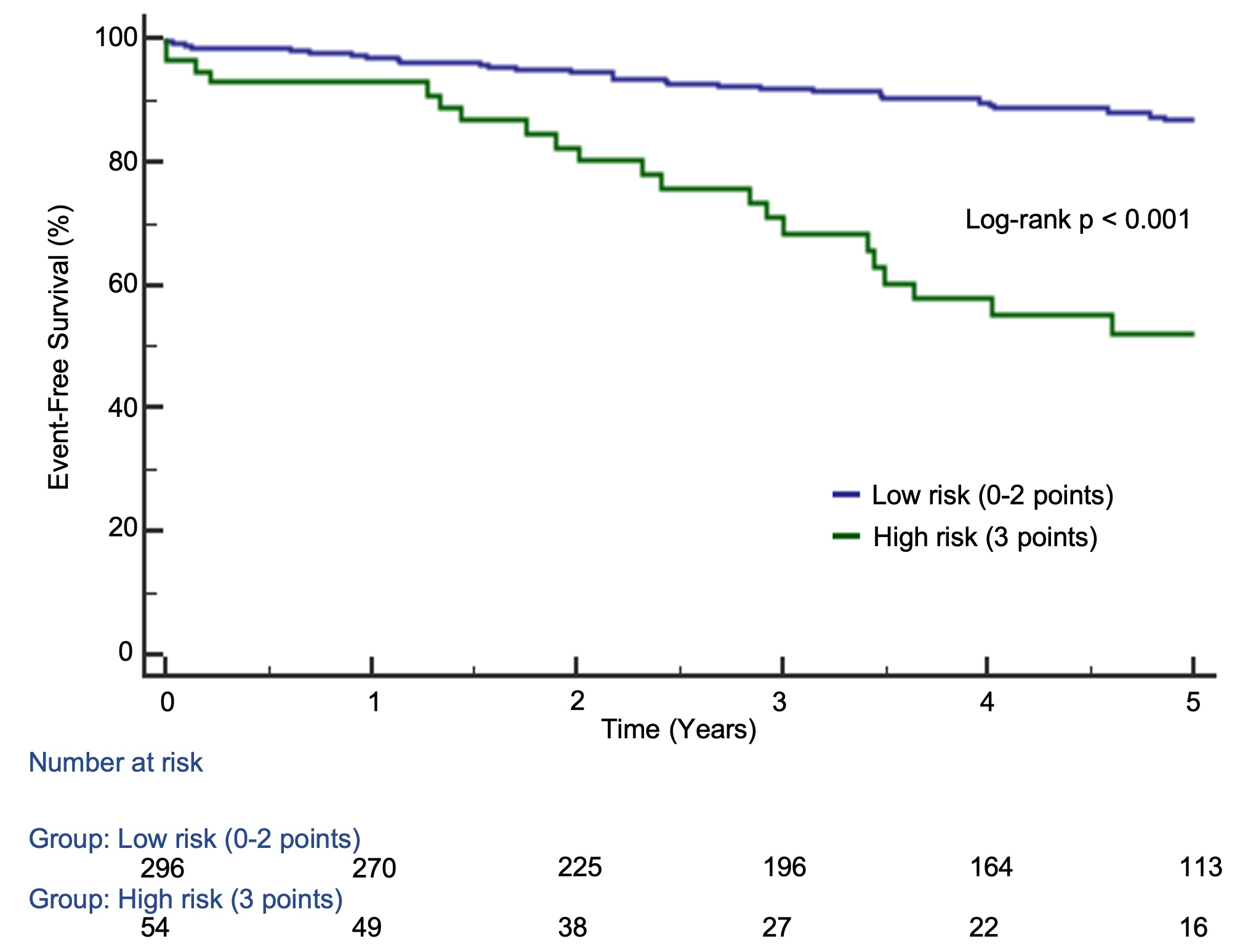


**Figure *2*. Kaplan-Meier analysis of a simplified CMR risk score in DCM patients**. The simplified CMR risk score is based on LVEF, LGE and GLS as the single strain parameter for the prediction of the primary endpoint (cut-off values as mentioned in Figure 4 of the manuscript): low risk (0-2p) and high risk (3p).

CMR: cardiac magnetic resonance imaging; DCM: dilated cardiomyopathy; LGE: late gadolinium enhancement; LVEF: left ventricular ejection fraction; GLS: global longitudinal strain.
